# Ramadan Fasting in Kidney Transplant Recipients: A Single-Centre Retrospective Study

**DOI:** 10.1155/2018/4890978

**Published:** 2018-06-03

**Authors:** Ihab A. Ibrahim, Ehab A. Hassan, Abdelrahman M. Alkhan, Mohamed A. Hussein, Ahmed F. Alhabashi, Tariq Z. Ali, Yasir Z. Shah, Ibrahim S. Alahmadi, Mohamed S. Abdelsalam, Mohamed E. Rashwan, Ammar Abdulbaki, Dieter C. Broering, Hassan A. Aleid

**Affiliations:** ^1^Department of Kidney and Pancreas Transplantation, King Faisal Specialist Hospital and Research Center, Riyadh, Saudi Arabia; ^2^Division of Nephrology, Department of Internal Medicine, Cairo University School of Medicine, Cairo, Egypt; ^3^Division of Nephrology, Department of Internal Medicine, Fayoum University, Fayoum, Egypt; ^4^Division of Nephrology, Department of Internal Medicine, Alexandria University, Alexandria, Egypt; ^5^Organ Transplant Center, King Faisal Specialist Hospital and Research Center, Riyadh, Saudi Arabia

## Abstract

**Background:**

Fasting during the lunar month of Ramadan is mandatory to all healthy adult Muslims. Renal transplant recipients are often worried about the impact of fluid and electrolyte deprivation during fasting on the function of their allograft. We aimed to examine the effect of fasting Ramadan on the graft function in renal transplant recipients.

**Methods:**

This retrospective cohort study included patients who underwent kidney transplantation in our tertiary referral center. Baseline pre-Ramadan estimated glomerular filtration rate (eGFR), mean arterial pressure (MAP), and urinary protein excretion were compared to those during and after Ramadan within and between the fasting and non-fasting groups.

**Results:**

The study population included 280 kidney transplant recipients who chose to fast during the Ramadan month (June-July 2014) and 285 recipients who did not fast. In the fasting group, baseline eGFR did not change from that during or post-Ramadan (72.6 ± 23.7 versus 72.3 ± 24.5 mL/min/1.73 m2, *P* = 0.53; and 72.6 ± 23.7 versus 72 ± 23.2 mL/min/1.73 m2, *P* = 0.14, respectively). Compared to baseline, there were no significant differences between the fasting and the non-fasting groups in terms of mean percent changes in eGFR, MAP, and urinary protein excretion.

**Conclusion:**

Fasting during the month of Ramadan did not have significant adverse effects on renal allograft function.

## 1. Introduction

Ramadan, the ninth lunar month of Islamic (Hijri) calendar, is a sacred month for Muslims all around the world. Fasting during the month of Ramadan is obligatory to all healthy adult Muslims [1].

During this month, Muslims refrain from eating, drinking, and taking medications from dawn to sunset each day. As the Islamic calendar is lunar, the beginning of the Islamic year moves ahead 11 days each year compared with the solar (Gregorian) year; thus, the month of Ramadan happens at various times of the seasonal year over a 33-year cycle [2, 3]. This can cause the Ramadan fast being observed in notably diverse environmental conditions between years in the same country [3]. Accordingly, the fasting hours can vary from 12 to 18 h, depending on the seasonal and regional characteristics [3].

Patients for whom fasting may be detrimental to health are exempted from fasting [1]. However, each year, many patients express their eagerness to observe the fast to respect the traditional customs [4].

The restoration of renal function and the enhancement of survival allow renal transplant recipients to share normal life activities including fasting. The question about the safety of fasting on renal allograft can be challenging for the healthcare professionals who need to be familiar with this spiritual commitment as well as its impact on health [5]. The major concern in transplant patients is that dehydration, and the subsequent renal hypoperfusion during fasting may result in deterioration in renal function or facilitate rejection episodes [6, 7].

There have been a few studies that have previously assessed the effect of Ramadan fasting on patients with renal transplantation, with normal or moderately impaired renal function [7–15]. Although these studies have demonstrated that fasting is safe for kidney transplant recipients with respect to renal allograft function [7–15], the overall number of transplant patients was not large enough to have a reliable conclusion about safety.

Here, we report on one of the largest cohort studies conducted to examine the potential effect of fasting during the month of Ramadan, on renal function in kidney transplant recipients with normal or impaired graft function.

## 2. Patients and Methods

### 2.1. Study Design and Setting

An observational retrospective cohort study was conducted in a tertiary referral centre in Riyadh, Saudi Arabia, by reviewing patients' records from the Renal Transplant Clinic.

### 2.2. Sampling and Study Participants

We recruited our patients through a written questionnaire (linked with patient record review), given to patients who presented for follow-up at the Renal Transplant Clinic from July 1, 2015, to December 30, 2015. The questionnaire included whether they observed the fast or not, the advice given by the health educator prior to fasting concerning fluids and medications intake, and the development of any complications during fasting. The source population included all kidney transplant recipients, aged >18 years, living in Riyadh (same environmental conditions), who had stable renal graft function for at least 6 months and transplant duration for more than one year prior to the month of Ramadan, corresponding to June/July 2014. We excluded patients who had been living outside the region of Riyadh to exclude the climate discrepancy bias. The sample size was calculated using the STATCALC module of Epi-Info v.7 software. Estimation was based on assumptions with a power of 80%, *α* (type I) error of 5%, 25% proportion of outcome among exposed (fasting), risk ratio (RR) of 1.66 with 0.5 standard deviation (SD), and lost to follow-up (missing data) rate of 5%. Based on the above assumptions, the total sample size included 602 patients with exposed (fasting) to non-exposed (non-fasting) ratio of 1 : 1. Non-probability consecutive sampling method was used to sample individuals into the two groups (fasting and nonfasting) from the eligible patients in the records. Patients with incomplete baseline and/or outcome data were excluded. The final cohort included 565 patients with complete data for analysis. The data of patients who had voluntarily chose to observe fast during the Ramadan month were analyzed and compared to those of patients who had not engaged in fasting. The study followed the STROBE reporting guidelines [16].

### 2.3. Data Sources and Management

A standard data collecting checklist was prepared based on routine data registration using standardized care and follow-up forms employed by the Renal Transplant Clinic in our institution. Data were extracted from patients' files and electronic medical records by eight adult kidney transplant staff physicians using the prepared data collecting checklist. Extracted demographic and clinical data of transplant recipients included age, gender, body mass index (BMI), primary kidney disease, type of donor (living donor, deceased donor), immunosuppressive protocols (drugs and doses), time since transplant, and presence or absence of diabetes mellitus and hypertension. The clinical and biochemical data of eligible patients were examined in three occasions: baseline pre-Ramadan (the last visit within 3 months before the month of Ramadan), during (after mid-Ramadan), and post-Ramadan (the first visit within 3 months after the month of Ramadan). The following parameters were analyzed: BMI; systolic, diastolic, and mean blood pressure; serum creatinine, eGFR, timed trough plasma levels of cyclosporine and tacrolimus; and urine protein excretion. The glomerular filtration rate (GFR) was estimated using a four-variable modification of diet in renal disease (MDRD) formula [17].

During the month of Ramadan, patients were advised by our health educators to regularly take the usual dose of their twice-daily medications (including immunosuppressive drugs) immediately at the time of breaking the fast at sunset, and just before re-starting their fast at dawn. The changes in estimated GFR and other parameters, from baseline to during and after the month of Ramadan, were calculated and compared for the fasting and non-fasting groups.

### 2.4. Outcomes

The primary outcome of this study was change in graft function (sCr and eGFR) from baseline pre-Ramadan to during and post-Ramadan values. The secondary outcomes were acute rejection rates and graft loss.

### 2.5. Ethical Considerations

The current study protocol was approved by our research ethics committee and institutional review board (IRB). The study was carried out in accordance with the ethical standards of the Declaration of Helsinki and the declaration of Istanbul. A written informed consent was obtained from each participant before questionnaire completion (for enrollment) and retrospective examination of their medical records (for data extraction).

### 2.6. Statistical Analysis

Data were analyzed using the statistical packages for social sciences (SPSS) version 20.0.0 (SPSS Inc., Chicago, IL, USA) for Microsoft Windows. Categorical variables were expressed as number and percentage, while continuous variables were expressed as mean ± standard deviation (SD). The *t*-test was used to assess the differences between the fasting and the non-fasting participants in terms of baseline characteristics and differences in eGFR. Chi-square (*χ*2) and Fisher exact test were used to find the association between two independent variables. The paired *t*-test was used to assess the differences between the pre- and post-Ramadan eGFR among the fasting participants. Analysis of variance (ANOVA) was used to compare means of variables in more than two groups (eGFR subgroups). We presented a forest plot to summarize the standardized mean differences (SMD) in eGFR of fasting versus non-fasting patients in subgroups including age, post-transplant time frames, presence of DM or HTN, baseline eGFR, and proteinuria. Multivariate linear regression analysis models were built to determine the independent variables significant as predictors of outcomes after adjustment for possible confounders. Results were considered significant when the two-sided *P* value was lower than 0.05.

## 3. Results

**Figure 1:**
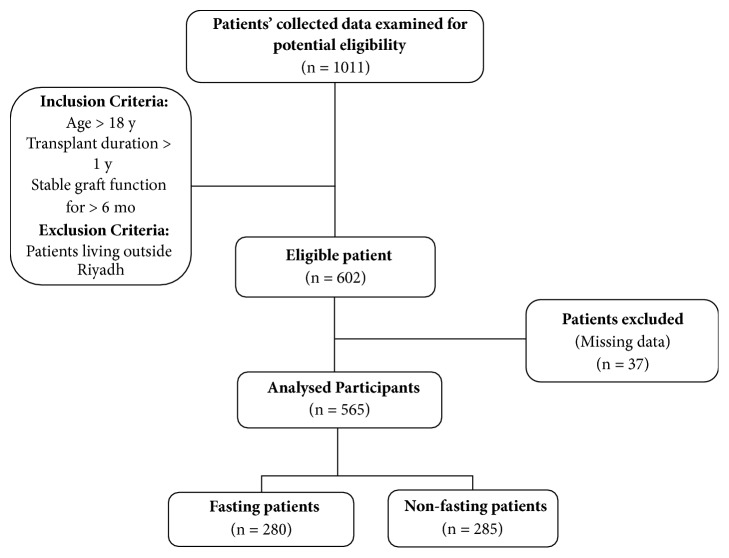
Flow chart illustrates subject enrollment and analysis. A total of 565 subjects were included for analysis in this retrospective cohort study.

A total of 1011 kidney transplant recipients completed the questionnaire. After patient record review and applying inclusion/exclusion criteria, 602 participants were left eligible. We further excluded 37 subjects with missing data (listwise deletion) on covariates or outcome data results (e.g., absence of serum creatinine measurements at any of the predefined study occasions). Thus, the final cohort included 565 participants with complete data. These were further subdivided into 2 groups, the fasting group (280 patients) and the non-fasting group (285 patients) (Figure 1)

During the month of Ramadan (corresponding to June/July 2014), the daily fasting hours ranged from 12 to 14 hours, the average temperature was 36°C, and the highest temperature during the day was 44°C [18], while the average humidity ranged from 14% to 16% [19].

The two groups were nearly comparable at baseline (Table 1), yet we chose to compare changes in renal function rather than absolute values. The fasting participants acted as their own controls in detecting the impact of fasting on allograft function.

The characteristics of both groups are shown in Table 1. The mean fasting duration was 29 ± 3.2 days (Range 15–30), with 260 patients in the fasting group completing fasting for the whole month while the other 20 did not because they experienced complications or decided not to continue. Complications included CNI toxicity (6 patients), urinary tract infection (8 patients), and other infections (chest infection, upper respiratory tract infection, and acute diarrheal illness) in 3 patients. These 20 patients (who fasted more than 14 days but less than 30 days) were included in the final analysis of the fasting group.

The two groups were similar in age, gender, body mass index, prevalence of coexisting diabetes and hypertension, type of transplant, immunosuppressive regimens, degree of proteinuria, baseline eGFR, and serum creatinine (SCr) (Table 1). The fasting group, however, had a longer mean duration since transplantation (81.9 ± 55.5 versus 67.18 ± 52.3 months, respectively; *P* = 0.001) (Table 1).

The mean time interval (months) from the baseline SCr/eGFR value to the start of Ramadan was different in both the fasting and the non-fasting groups (1.65 ± 0.57 versus 1.79 ± 0.67, *P* = 0.013) while the mean time interval from the end of Ramadan to the first SCr/eGFR after Ramadan was similar (1.49 ± 0.53 versus 1.45 ± 0.39, *P* = 0.3), as well as the time range interval (months) between the baseline pre-Ramadan and the follow-up post-Ramadan readings (4.14 ± 0.98 versus 4.24 ± 0.99, *P* = 0.27).

The mean blood pressure, renal function, urinary protein excretion, and drug levels pre-, during, and post-Ramadan are shown in Table 2. Despite some significant intra-group changes in post-Ramadan mean blood pressure and tacrolimus levels, compared to pre-Ramadan, no statistically significant difference in these parameters after Ramadan compared to that before Ramadan was noted between the two groups (*P* value > 0.05, Table 2). The mean serum creatinine levels before Ramadan were similar in both groups (*P*-value = 0.85), as were the mean eGFRs (*P*-value = 0.97). In the fasting group, the mean serum creatinine levels before and during Ramadan (107 ± 48.3 versus 107.7 ± 49.6 *μ*mol/L, with *P* value = 0.2) as well as before and after Ramadan (107 ± 48.3 versus 107.8 ± 49 *μ*mol/L, *P* value = 0.06) were similar, as were the eGFR values before and during Ramadan (72.6 ± 23.7 versus 72.3 ± 24.5 mL/min/1.73 m2, *P* = 0.53), and before and after Ramadan (72 ± 23.2 versus 72.6 ± 23.7 *μ*mol/L, *P* value = 0.14) (Table 2). Similarly, in the non-fasting group, no difference was seen between the baseline and during Ramadan or the baseline and post-Ramadan creatinine levels (*P* value = 0.9 and *P* = 0.45, respectively) or eGFR values (*P* = 0.07 and *P* value = 0.08, respectively) (Table 2).

When we compared the fasting group to the non-fasting group in terms of the mean percent changes in serum creatinine, eGFR, MAP, and proteinuria between baseline values and those after Ramadan, and after adjustment to other parameters including gender, age, transplant type, time since transplant, hypertension, diabetes, and baseline eGFR or proteinuria, we found no significant differences in eGFR (*P* = 0.7), MAP (*P* = 0.7), or proteinuria (*P* = 0.08) (Table 3).

A multiple linear regression analysis was performed to assess the correlation of demographic, clinical, and laboratory variables with the change in eGFR. Regression analysis displayed statistical significance for baseline eGFR (*P* < 0.0001) and baseline proteinuria (*P* = 0.032) with change in eGFR (Table 4).

**Figure 2:**
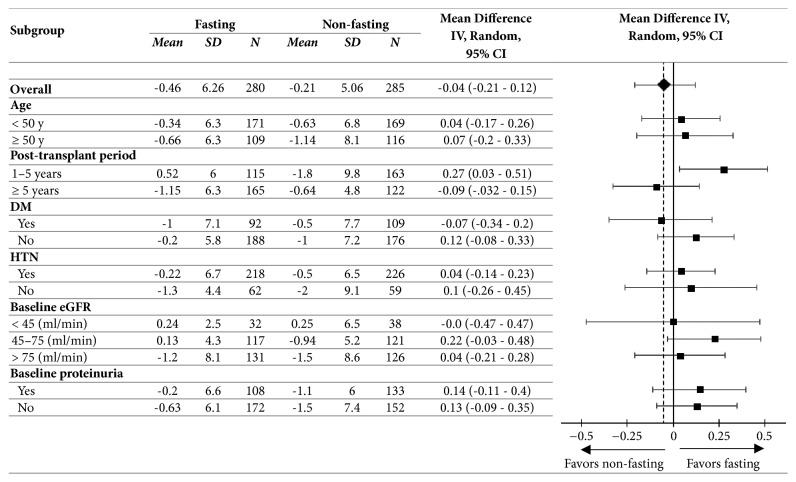
Forest Plot of Primary Outcome According to Subgroups. The dashed vertical line represents the mean difference for the overall study population.

A forest plot of the standardized mean difference (SMD) measure of effect summarized exploratory subgroup analyses for change in eGFR in fasting versus non-fasting patients (Figure 2). The subgroups included age categories (less or more than 50 years), post-transplant time frames (1 to 5 years and more than 5 years), presence of DM or HTN, baseline eGFR and proteinuria. The result of subgroup analyses indicated that the effect size (SMD) was consistent across the prespecified subgroups with respect to the primary outcome.

As some patients with renal allograft impairment decided to observe the fast, we further divided the fasting group into three subgroups, according to baseline eGFR, low (<45 mL/min/1.73 m2), moderate (45–75 mL/min/1.73 m2), and high GFR (>75 mL/min/1.73 m2). Statistical analysis revealed no significant differences in the eGFR after Ramadan compared to that at baseline, within or between the subgroups (*P* value > 0.05) (Table 5).

The frequency of acute adverse events during the month of Ramadan in both fasting and non-fasting groups was not statistically different. CNI toxicity was detected in 6 (2%) versus 8 (2.8%) patients, respectively (*P* = 0.53); urinary tract infection was detected in 8 (2.9%) versus 8 (2.8%) patients, respectively (*P* = 0.97); and other infections (chest infection, upper respiratory tract infection, and acute diarrheal illness) in 3 (1%) versus 6 (2.1%) patients, respectively (*P* = 0.33). No rejection episodes or renal function deterioration were noted after Ramadan. There were no graft or patient losses in any of the groups.

Also, baseline pre-Ramadan values of other biochemical and metabolic parameters were compared to those during and after Ramadan within and between the fasting and non-fasting groups, and no statistically significant difference was noted (*P* value > 0.05, Table S1 and S2)

## 4. Discussion

In our study, we found no significant difference in eGFR during or after Ramadan from that at baseline in the fasting group. When we compared the fasting group to the non-fasting group in terms of the mean percent changes in eGFR, MAP, and proteinuria between baseline and after Ramadan, we found no significant differences.

Ramadan fasting represents one of the five pillars of the Islamic religion. Even though patients are exempted from observing this religious duty, they may be keen to share this spiritual moment of the year with their family and peers. However, there are no evidence-based guidelines or standardized protocols that address the issue of renal transplant patients fasting in Ramadan and correctly guide and counsel them. Muslims constitute 1.8 billion (24.1%) of the world's population [20] and inhabit all corners of the globe; thus studying this issue is of paramount importance to healthcare professionals taking care of these patients. To our knowledge, this is the largest observational study exploring the effect of fasting Ramadan on the graft function in kidney transplant patients.

Daytime dehydration due to water deprivation during the intermittent fast of Ramadan will induce a degree of stress on the concentrating ability of the kidneys [21]. The major concern in transplant patients is that dehydration and accumulation of metabolites may result in deterioration in renal function or facilitate rejection episodes [21]. Previous studies demonstrated a preserved renal concentrating ability in renal transplant recipients with stable allograft function after a daylong Ramadan fast [8, 22]. No detrimental effects on kidney function have yet been directly attributed to intermittent negative water balance at the levels that may be produced during Ramadan [21].

There have been a few studies that have previously assessed the effect of Ramadan fasting on renal transplant patients with normal or impaired renal function [7–15]. These studies included fasting Ramadan at various times of the year (cold and hot months), only one-time, and repeated Ramadan fasting and some included control non-fasting groups [7–15]. In general, these studies have demonstrated that Ramadan fasting is safe for kidney transplant recipients with respect to renal allograft function and other biochemical parameters [7–15] and our study further strengthens this observation, which is important as randomized trials to prove safety of Ramadan fasting on renal allograft function are not feasible.

As we divided the fasting group into three subgroups, according to baseline eGFR, low (<45 mL/min/1.73 m2), moderate (45–75 mL/min/1.73 m2), and high GFR (>75 mL/min/1.73 m2), we found no significant differences in the eGFR after Ramadan from that at baseline, within or between the subgroups (*P* value > 0.05) (Table 5). These findings are also consistent with earlier reports of safety of Ramadan fasting in patients with different degrees of renal impairment [11–15]. In a previous report, Einollahi et al. [11] investigated the impact of Ramadan fasting on 41 kidney transplant recipients who decided to fast during Ramadan and compared their findings with 41 matched control recipients who did not fast. The authors found no negative impact on graft function following fasting during Ramadan in kidney recipients who had normal as well as mild to moderate impaired (eGFR < 60 mL/min) but stable renal allograft function prior to fasting [11]. In another study, Boobes et al. [12] investigated the impact of Ramadan fasting on 22 kidney transplant recipients with stable kidney functions who had undergone renal transplantation more than 1 year prior to Ramadan. The authors concluded that it is safe for kidney transplant recipients of more than one year and stable graft function to fast Ramadan. However, they cautioned about possible adverse impact of fasting on the patients with moderate to severe impaired renal function despite nonspecific findings [12]. Qurashi et al. [13] investigated the impact of Ramadan fasting on 43 renal transplant recipients who voluntarily decided to fast during Ramadan and compared them with 37 matched controls who did not fast. They found that fasting Ramadan during the hottest month of the year (August) did not adversely affect graft function even after a follow-up period of 6 months [13]. The effect of repeated Ramadan fasting on 35 kidney transplant recipients was investigated by Ghalib et al. [14] They found no significant changes in the eGFR after fasting for three consecutive Ramadan months. In a similar study, Hejaili et al. [15] found that fasting during two consecutive Ramadan months in Riyadh did not adversely affect graft function even after a follow-up period of over 19 months [15].

In our study, we found no significant difference in the frequency of acute complications during Ramadan in both the fasting and the non-fasting groups. No deterioration in renal function or rejection episodes were observed during or after Ramadan. There were no graft or patient losses in any of the groups. These findings agree with the results of other reports [8–15].

When interpreting our study, some* limitations* need to be considered. The study cohort is representative for a Western Asian, primarily Arab, population and results might thus not be generalizable to populations in other regions of the world or with different ethnic backgrounds. Despite multivariate adjustment to reduce bias, our findings might still be affected by residual confounders, either unknown or unmeasured (e.g., seasonality, variability in daily fasting time, physical activity, smoking, medications, and eating pattern), which cannot be ruled out in any observational study [23]. Further, the impact of fasting on the renal tubular function and the immune system was not directly assessed. Finally, we did not consider the causes of non-fasting which might infer selection bias (e.g., patients who observed the fast may be of better health status compared to those who did not observe Ramadan fasting who might be elderly or debilitated or have multiple comorbidities), but the two groups were comparable at baseline (Table 1).

Our study has several* strengths*, first and foremost, the availability of outcome data of high quality in a well-maintained electronic medical record. Furthermore, we have a large sample size of several hundred transplant recipients with a sufficiently substantial number of patients to conduct adequate analyses, while the existing studies collectively included fewer than 400 participants.

## 5. Conclusion

Based on the results of this study, we believe that fasting during Ramadan has no adverse effects on renal function in kidney transplant recipients with normal as well as impaired renal allograft function. We think fasting can be allowed when the renal graft function has been stable for at least 6 months and the transplanted kidney has been in place for at least one year. It is recommended, however, that all renal transplant patients, who choose to fast, to do so under medical supervision and to pay strict attention to fluid intake, daily physical activity, drug regimen adjustment, and renal function monitoring. The patients should be advised to drink liberal amounts (at least 2.5 L) of fluids after breaking their fast (immediately after sunset), till just before the start of the fast (predawn) and to avoid excessive exposure to heat or overexertion to avoid dehydration.

Despite the fact that Ramadan fasting proved safe across all eGFR levels in our study, the definition of a specific allograft function values (GFR level) as safe for Ramadan fasting still needs longer follow-up analyses.

With the currently available data, there is no evidence of substantial graft injury associated with fasting; however, the possibility of a long-term adverse renal outcome cannot be excluded. These observations and hypotheses need to be explored further in analyses that incorporate more clinical outcomes and longer follow-up.

## Figures and Tables

**Table 1:** Basic demographic and clinical data in fasting and non-fasting groups^a,b^.

Parameter	Fasting (*N* = 280)	Non-fasting (*N* = 285)	*P* value^*∗*^
*Age (Y)*			
Overall (mean ± SD)	44.2 ± 15.8	43.6 ± 16.2	0.66
>50 y, *n* (%)	101 (36.1)	107 (37.5)	0.73
*Sex*			
Male, *n* (%)	178 (63.57)	176 (61.75)	0.65
Female, *n* (%)	102 (36.43)	109 (38.25)	
*BMI (kg/m* ^*2*^ *) (mean ± SD)*	28.62 ± 7.6	28.36 ± 7.04	0.67
*Original kidney disease*, *n* (%)			
DM	61 (21.8)	71 (24.9)	0.85
HTN	20 (7.1)	14 (4.9)	
GN	52 (18.6)	49 (17.2)	
Others	147 (52.5)	151 (53)	
*Type of donor, n* (%)			
LD	229 (81.8)	232 (81.4)	0.97
DD	51 (18.2)	53 (18.6)	
*Post-transplant period (months)* (mean ± SD)	81.87 ± 55.5	67.18 ± 52.3	**0.001**
*DM*, *n* (%)	92 (33)	109 (38.4)	0.19
*HTN*, *n* (%)	218 (78)	226 (79.3)	0.68
*Baseline eGFR (ml/min/1.73 m* ^*2*^)			
Overall (mean ± SD)	72.74 ± 23.54	72.8 ± 25.8	0.97
<45, *n* (%)	32 (11.4)	38 (13.3)	0.87
45–75, *n* (%)	117 (41.8)	121 (42.5)	
>75, *n* (%)	131 (46.8)	126 (44.2)	
*Baseline SCr (umol/l)*	106.6 ± 48.5	107.4 ± 47	0.85
*Baseline proteinuria (g/day)*			
Yes, *n* (%)	108 (38.6)	133 (46.7)	0.052
No, *n* (%)	172 (61.4)	152 (53.3)	

^a^ *Abbreviations*. BMI, body mass index; DM, diabetes; HTN, hypertension; GN, glomerulonephritis; LD, living donor; DD, deceased donor; SCr, serum creatinine; eGFR, estimated glomerular filtration rate. ^b^Data are presented as number (percentage) or mean ± SD; ^*∗*^*P* value is significant if <0.05.

**Table 2:** Comparing changes in renal function and other parameters between baseline values and during and after Ramadan in fasting and non-fasting groups^a,b^.

Parameter	Pre-Ramadan	During Ramadan	Post-Ramadan	Δ pre – during (*P* value^$^)	Δ pre – post (*P* value^$^)
MAP (mmHg)					
F	93.2 ± 10	93.3 ± 8.4	95 ± 10.5	0.9	0.002
NF	93 ± 11	93.2 ± 9	94.8 ± 10.77	0.63	0.001
*P* *value*^*∗*^	0.76	0.9	0.95	0.67^∧^	0.72^∧^
sCr (umol/l)					
F	107 ± 48.3	107.7 ± 49.6	107.8 ± 49	0.2	0.06
NF	107.4 ± 47	107.5 ± 42.7	108.2 ± 44	0.9	0.45
*P* *value*^*∗*^	0.9	0.96	0.9	0.67^∧^	0.33^∧^
eGFR (ml/min)					
F	72.6 ± 23.7	72.3 ± 24.5	72 ± 23.2	0.53	0.14
NF	72.8 ± 25.8	72 ± 25	72.1 ± 25.1	0.07	0.08
*P* *value*^*∗*^	0.9	0.85	0.8	0.95^∧^	0.7^∧^
Protein excretion (g/day)					
F	0.47 ± 1.17	0.44 ± 1.03	0.50 ± 1.12	0.28	0.19
NF	0.48 ± 1.18	0.47 ± 1.16	0.52 ± 1.26	0.12	0.085
*P* *value*^*∗*^	0.75	0.75	0.6	0.65^∧^	0.08^∧^
Tacrolimus level					
F	6.2 ± 1.9	6.4 ± 2.1	6.8 ± 2.4	0.015	0.0001
NF	6.5 ± 2	6.6 ± 1.9	6.6 ± 1.8	0.43	0.11
*P* *value*^*∗*^	0.08	0.43	0.47	0.83^∧^	0.044^∧^

^a^ *Abbreviations*. MAP, mean arterial pressure; sCr, serum creatinine; eGFR, estimated glomerular filtration rate. ^b^Data are presented as mean ± SD; *P* value is significant if <0.05; ^*∗*^*P* value in-between groups; ^$^*P* value within each group; ^∧^*P* value of the mean percent change of each parameter during and after Ramadan compared to before Ramadan in the fasting versus non-fasting groups.

**Table 3:** Changes in eGFR, creatinine, MAP, and protein excretion between baseline and after Ramadan^a,b^ in the fasting versus non-fasting groups.

Parameter	Fasting (*n* = 280)	Non-fasting (*n* = 285)	Adjusted *P* value^*∗*^
Change in post-Ramadan eGFR (ml/min) (mean ± SD)	0.56 ± 6.4	1.3 ± 8	0.23
% Change in post-Ramadan eGFR (mean ± SD)	0.32 ± 7.9	0.73 ± 15.6	0.7
Change in post-Ramadan sCr (umol/l) (mean ± SD)	0.9 ± 7.9	0.87 ± 19.7	0.99
% Change in post-Ramadan sCr (mean ± SD)	1.13 ± 7.9	1.83 ± 9.7	0.33
Change in MAP (mmHg) (mean ± SD)	1.74 ± 9.6	1.96 ± 9.7	0.8
% Change in MAP (mean ± SD)	2.4 ± 10.6	2.7 ± 10.8	0.7
Change in Proteinuria (g/day) (mean ± SD)	0.04 ± 0.5	0.06 ± 0.6	0.6
% Change in Proteinuria (mean ± SD)	57.3 ± 142	37.7 ± 120	0.08

^a^ *Abbreviations*. MAP, mean arterial pressure; Scr, serum creatinine; eGFR, estimated glomerular filtration rate; ^b^Data are presented as mean ± SD; ^*∗*^*P* value is significant if <0.05; *P* values were adjusted for sex, age, transplant type, hypertension, diabetes, and duration since transplantation.

**Table 4:** Multivariate linear regression model of selected predictor variables and change in renal function (change in eGFR).

Predictor variables	Standardized coefficient (*β*)	Standard error	*t*	Significance (CI)
Age	0.026	0.042	0.628	0.531 (−0.056, 0.108)
Post-transplant period	−0.031	0.043	−0.724	0.469 (−0.116, 0.053)
MAP	−0.006	0.041	−0.146	0.884 (−0.087, 0.075)
DM	0.023	0.043	0.521	0.603 (−0.062, 0.107)
HTN	−0.072	0.043	−1.65	0.098 (−0.156,0.013)
Baseline eGFR	−0.24	0.042	−5.74	**<0.0001 (**−**0.323, **−**0.158)**
Baseline proteinuria	−0.09	0.042	−2.15	**0.032 (**−**0.172, **−**0.008)**

*R* ^2^ = 0.06, Adjusted *R*^2^ = 0.053, Mean Squared Error (MSE) = 43				

^a^ *Abbreviations*. MAP, mean arterial pressure; DM, diabetes; HTN, hypertension; eGFR, estimated glomerular filtration rate; ^b^*P* value is significant if <0.05.

**Table 5:** Comparing changes in renal function among fasters with different baseline GFR^a,b^.

Parameter	Low baseline eGFR group (<45 ml/min)	Moderate baseline eGFR group (45–75 ml/min)	High baseline eGFR group (>75 ml/min)	*P* value^*∗*^
*N* (%)	32 (11.4%)	117 (41.8%)	131 (46.8%)	0.001
Age (Y)	40.9 ± 15	42.6 ± 15.2	46.3 ± 16.2	0.08
Gender (Male/Female)	21/11	76/40	81/51	0.75
BMI (kg/m^2^) (mean ± SD)	28.5 ± 9.7	29.2 ± 6.6	28.2 ± 7.9	0.55
SBP (mmHg)	128.4 ± 14	126 ± 16	125.8 ± 14.1	0.66
DBP (mmHg)	79 ± 12	77 ± 10.8	76.12 ± 9.6	0.35
MAP (mmHg)	97.34 ± 11	91.8 ± 10	93.4 ± 9.7	0.02
Mean baseline eGFR	33.2 ± 9	62 ± 9.6	91.5 ± 16	0.0001
Mean eGFR after Ramadan	33.5 ± 9.8	62 ± 9.7	90.2 ± 16.2	0.0001
Mean change in eGFR (ml/min)	0.24 ± 2.46	0.11 ± 4.3	−1.4 ± 8.24	0.14
Mean % change in eGFR	0.42 ± 6.8	0.45 ± 7.2	−1.2 ± 8.6	0.23
*P* value of change within each group	0.6	0.77	0.06	

^a^ *Abbreviations*. BMI, body mass index; SBP, systolic blood pressure; DBP, diastolic blood pressure; MAP, mean arterial pressure; eGFR, estimated glomerular filtration rate. ^b^Data are presented as number (percentage) or mean ± SD; ^*∗*^*P* value is significant if <0.05.

## Data Availability

All data arising from this study are contained within the manuscript and supplementary material files.

## Abbreviations

ANOVA: analysis of variance
BMI: body mass index
CNI: calcineurin inhibitors
DBP: diastolic blood pressure
DD: deceased donor
DM: diabetes
eGFR: estimated glomerular filtration rate
GN: glomerulonephritis
HTN: hypertension
IRB: institutional review board
LD: living donor
MAP: mean arterial pressure
MDRD: modification of diet in renal disease
SBP: systolic blood pressure
SMD: standardized mean differences
sCr: serum creatinine
SD: standard deviation
SPSS: statistical packages for social sciences.

## Supplementary Materials

Supplementary Materials Additional file: Spreadsheet containing the demographic and clinical data of the study groups. Table (S1): comparing changes in biochemical and metabolic parameters between pre-Ramadan (baseline) and during Ramadan in fasting and non-fasting groups. Table (S2): comparing changes in biochemical and metabolic parameters between pre-Ramadan (baseline) and post-Ramadan in fasting and non-fasting groups.
