# Supplementary material for: Ramadan Fasting in Kidney Transplant Recipients: A Single-Centre Retrospective Study
Source: J Transplant. 2018 Jun 3;2018:4890978. doi: 10.1155/2018/4890978 (PMC6008647; doi:10.1155/2018/4890978)
Supplement: Supplementary Materials — Additional file: Spreadsheet containing the demographic and clinical data of the study groups. Table (S1): comparing changes in biochemical and metabolic parameters between pre-Ramadan (baseline) and during Ramadan in fasting and non-fasting groups. Table (S2): comparing changes in biochemical and metabolic parameters between pre-Ramadan (baseline) and post-Ramadan in fasting and non-fasting groups. [file 4890978.f1.pdf]

Table (S1): Comparing changes in biochemical and metabolic parameters between pre-Ramadan (baseline) and during Ramadan in fasting and non-fasting groups[a,b]

Parameter		Pre-Ramadan	During Ramadan	Δ pre – during (p value[$])
**Na (mmol/l)**	***F***	**141.6±3.5**	**140.2±3**	**0.0001**
	NF	**141.7±2.9**	**140.1±2.7**	**0.0001**
	P value	**0.75**	**0.8**	**0.454[^]**
**K (mmol/l)**	***F***	**4.4±0.4**	**4.5±0.4**	**0.006**
	NF	**4.4±0.4**	**4.4±0.4**	**0.03**
	P value	**0.8**	**0.5**	**0.7[^]**
**HCO3 (mmol/l)**	***F***	**23.9±2.9**	**24.4±3.2**	**0.005**
	NF	**24±3**	**24.2±3**	**0.26**
	P value	**0.6**	**0.4**	**0.2[^]**
**Albumin (g/l)**	***F***	**42±3.9**	**43±3.7**	**0.0001**
	NF	**42±3.8**	**42.5±3.9**	**0.004**
	P value[*]	**0.8**	**0.2**	**0.2[^]**
**Hb (g/l)**	***F***	**137±17.5**	**137.2±16.9**	**0.75**
	NF	**135.5±19**	**136.2±18**	**0.12**
	P value	**0.3**	**0.5**	**0.38[^]**
**WBCs**	***F***	**7±2**	**7.3±2.2**	**0.02**
	NF	**7.3±2.1**	**7.5±2.3**	**0.07**
	P value	**0.1**	**0.2**	**0.9[^]**
**Glucose**	***F***	**6.2±2.2**	**5.9±1.8**	**0.02**
	NF	**6.2±2.5**	**6±1.9**	**0.06**
	P value	**0.9**	**0.8**	**0.58[^]**
**T. Chol**	***F***	**4.2±0.8**	**4.2±0.9**	**0.0001**
	NF	**4.2±0.9**	**4.1±0.9**	**0.001**
	P value	**0.6**	**0.7**	**0.53[^]**
**HDL**	***F***	**1.23±0.3**	**1.3±0.4**	**0.0001**
	NF	**1.2±0.35**	**1.3±0.36**	**0.0001**
	P value	**0.9**	**0.65**	**0.56[^]**
**LDL**	***F***	**2.6±0.7**	**2.4±0.6**	**0.0001**
	NF	**2.6±0.7**	**2.5±0.65**	**0.0001**
	P value	**0.9**	**0.85**	**0.43[^]**
**TGs**	***F***	**1.5±0.7**	**1.53±0.8**	**0.3**
	NF	**1.5±0.74**	**1.54±0.84**	**0.1**
	P value	**0.99**	**0.9**	**0.97[^]**

[a]**Abbreviations**: Na, sodium; K, potassium; HCO_3, bicarbonate; Hb, hemoglobin; WBCs, white blood cells; T. Chol, total cholesterol; HDL, high-density lipoprotein; LDL, low-density lipoprotein; TGs, triglycerides; F, fasting; NF, non-fasting

[b]Data are presented as mean ± SD; p-value is significant if <0.05.

[*]P value in-between groups

[$] P value within each group

[^]P value of the mean **percent** change of each parameter during Ramadan compared to pre-Ramadan in the fasting versus nonfasting groups

Table (S2): Comparing changes in biochemical and metabolic parameters between pre-Ramadan (baseline) and post-Ramadan in fasting and non-fasting groups[a,b]

Parameter		Pre-Ramadan	Post-Ramadan	Δ Pre – post (p value[$])
**Na (mmol/l)**	***F***	**141.6±3.5**	**139.6±3.4**	**0.0001**
	NF	**141.7±2.9**	**139.5±3**	**0.0001**
	P value	**0.75**	**0.6**	**0.3[^]**
**K (mmol/l)**	***F***	**4.4±0.4**	**4.3±0.35**	**0.008**
	NF	**4.4±0.4**	**4.4±0.37**	**0.16**
	P value	**0.8**	**0.47**	**0.4[^]**
**HCO3 (mmol/l)**	***F***	**23.9±2.9**	**24±3.6**	**0.6**
	NF	**24±3**	**24.1±3.9**	**0.45**
	P value	**0.6**	**0.57**	**0.8[^]**
**Albumin (g/l)**	***F***	**42±3.9**	**41.4±3.9**	**0.0001**
	NF	**42±3.8**	**41.6±4.3**	**0.033**
	P value*	**0.8**	**0.6**	**0.4[^]**
**Hb (g/l)**	***F***	**137±17.5**	**137.2±18**	**0.77**
	NF	**135.5±19**	**136.2±20**	**0.2**
	P value	**0.3**	**0.54**	**0.47[^]**
**WBCs**	***F***	**7±2**	**7.3±2.5**	**0.04**
	NF	**7.3±2.1**	**7.6±2.6**	**0.02**
	P value	**0.1**	**0.16**	**0.8[^]**
**Glucose**	***F***	**6.2±2.2**	**6.3±2.3**	**0.37**
	NF	**6.2±2.5**	**6.3±2.5**	**0.2**
	P value	**0.9**	**0.9**	**0.78[^]**
**T. Chol**	***F***	**4.2±0.8**	**4.4±0.95**	**0.0001**
	NF	**4.2±0.9**	**4.3±1**	**0.0001**
	P value	**0.6**	**0.35**	**0.5[^]**
**HDL**	***F***	**1.23±0.3**	**1.3±0.4**	**0.0001**
	NF	**1.2±0.35**	**1.27±0.4**	**0.0001**
	P value	**0.9**	**0.8**	**0.66[^]**
**LDL**	***F***	**2.6±0.7**	**2.6±0.7**	**0.08**
	NF	**2.6±0.7**	**2.6±0.7**	**0.26**
	P value	**0.9**	**0.56**	**0.5[^]**
**TGs**	***F***	**1.5±0.7**	**1.55±0.8**	**0.06**
	NF	**1.5±0.74**	**1.54±0.84**	**0.2**
	P value	**0.99**	**0.85**	**0.72[^]**

[a]**Abbreviations**: Na, sodium; K, potassium; HCO_3, bicarbonate; Hb, hemoglobin; WBCs, white blood cells; T. Chol, total cholesterol; HDL, high-density lipoprotein; LDL, low-density lipoprotein; TGs, triglycerides; F, fasting; NF, non-fasting
[b]Data are presented as mean ± SD; p-value is significant if <0.05.
[*]P value in-between groups
[$] P value within each group
[^]P value of the mean **percent** change of each parameter post-Ramadan compared to pre-Ramadan in the fasting versus non-fasting groups
